# Editorial: Understanding Barriers to Workplace Equality: A Focus on the Target's Perspective

**DOI:** 10.3389/fpsyg.2020.01279

**Published:** 2020-06-17

**Authors:** Michelle K. Ryan, Christopher T. Begeny, Renata Bongiorno, Teri A. Kirby, Thekla Morgenroth

**Affiliations:** ^1^Department of Psychology, University of Exeter, Exeter, United Kingdom; ^2^Faculty of Economics and Business, University of Groningen, Groningen, Netherlands

**Keywords:** gender equality, workplace inequality, discrimination, work discrimination, intersectionality

The workplace continues to be the site of many group-based inequalities, including unequal rates of participation (e.g., Acker, 2006), biased rewards structures such as promotions and pay gaps (e.g., Metcalf, 2009), and discriminatory treatment on a day-to-day basis (e.g., Reskin, 2000). Barriers to achieving workplace equality can be overt or subtle; direct or indirect; reside in the workplace itself, or within society more broadly; and affect people from a range of social groups. Existing research has tended to focus on inequality based on gender (e.g., Barreto et al., 2009; Heilman, 2012) or people's racial or ethnic background (e.g., Sørensen, 2004). But there are also substantive bodies of literature examining inequality faced by LGBTQ+ individuals (e.g., Ragins and Cornwell, 2001; Hebl et al., 2002), older or younger employees (Diekman and Hirnisey, 2007), pregnant women and parents (e.g., Morgenroth and Heilman, 2017; Gloor et al., 2018), those with disabilities (McLaughlin et al., 2004), and those at the intersection of these and other identities (e.g., Ortiz and Roscigno, 2009; Holvino, 2010; Tatli and Özbilgin, 2012).

With this Research Topic we have two key aims: (1) to gain a better understanding of workplace inequality by defining it in a way that is inclusive of all potential forms of group-based discrimination; and (2) to focus on the target's perspective: those who are subjected to discriminatory treatment in the workplace, rather than on those who perpetuate inequality.

We have brought together 11 papers that address these key aims. We deliberately include research that examines a range of inequalities, including those based on gender, race, age, LGBTQ+ identities, weight stigma, and their intersections. In doing so, these papers cover a variety of workplaces, including those of PhD students and post-doctoral researchers in academia; humanitarian aid workers; and those working in STEM fields. The papers also examine issues of leadership succession and perceptions of leadership potential; mental health and health programs in the workplace; and mobilizing support for collective action. We have chosen authors that take a number of different approaches to researching inequality, including comprehensive reviews of the literature as well as empirical work encompassing qualitative interviews, experimental studies, and field studies.

We have two papers that examine issues of inequality within an academic context. Ysseldyk et al. focus on the mental health of women at the post-doctoral stage of their careers. Using a mixed methods approach they explore how identity development and lack of control contribute to the loss of women from academia. Their findings demonstrate the stress and tenuousness experienced by post-doctoral women and also highlight the potential protective factor of disciplinary identity. Looking at the start of the academic career pipeline, with a focus on PhD students, Bentley et al. examine the psychological mechanisms underlying identity construction and how it may contribute to inequality in career outcomes. They demonstrate that the perceived compatibility of one's identity with those of leading members of the profession can play an important role in on-going professional identity construction and career success.

Further building on issues of fit, two papers examine how perceptions of fit influence leadership processes more specifically. With a focus on the persistence of the “old boys club,” Rink et al. examine how interpersonal fit influences leaders' perceptions of their followers' potential as successors. Across two studies their results demonstrate that while male leaders ratings of followers' potential as successors were positively related to interpersonal fit (the old boys club), these relationships were absent for female leaders, suggesting there is not a comparable “old girls club.” Tresh et al. examine the role of gender and age stereotypes and their effect on individuals' job-related perceptions. Across three studies, results suggest that both gender and age stereotypes, and their incongruency with job roles, affect perceptions of: (a) leadership potential; and (b) job fit, and job appeal. This was particularly the case for older workers and for women. The paper also clearly demonstrates the importance of intersectional identities.

We include two papers that look specifically at issues of inclusion. Sahin et al. make an important distinction between employees' perceptions of deep- and surface-level similarity. They found that perceptions of deep-level dissimilarity to others in the workplace was associated with lower felt inclusion, while surface-level similarity did not affect felt inclusion. Rengers et al. examine perceptions of workplace inclusion in a field study of lesbian and gay humanitarian aid workers. Their findings revealed that perceptions of authenticity, but not belonging, are affected by the inclusiveness practices of their organization and by their colleagues' and supervisors' attitudes and behaviors. The authors construct a typology of three groups: conscious first-missioners, authentic realists, and idealistic activists.

In another field study, van Veelen et al. examine the effects of numerical and normative male dominance in STEM. They examine the impacts of women in STEM fields being outnumbered and negatively stereotyped. This “double trouble” predicted the highest levels of gender identity threat for women, which in turn, negatively impacted on their work engagement and career confidence. This was particularly the case for women with high levels of gender identity. In their theoretical paper, van Laar et al. further examine negative stereotypes across multiple groups. Using insights from research into stigma, social identity, and self-regulation, they provide a model for how individuals are affected by, and how they regulate, negative stereotypes in the workplace. They highlight four key processes: (1) the subtle triggers of workplace identity threat; (2) how individuals can cope with these threats; (3) factors that mitigate threat and affect self-regulation; and (4) the hidden costs of self-regulation.

We include two papers that focus on workplace approaches to promoting equality. Gündemir et al. provide a review of how diversity ideologies may affect self-perceptions and workplace experiences. They demonstrate that for members of racial-ethnic minority groups, ideologies that are diversity aware (e.g., multiculturalism) are more beneficial than ideologies that are diversity blind (e.g., colorblindness). In contrast, for women, gender blindness is associated with more positive outcomes than gender awareness. Täuber et al. examine potential downsides of workplace health promotion programs. Across three studies they demonstrate that health promotion programs can increase attributions of weight controllability, elicit weight stigma, and induce weight-based discrimination in workplace promotion decisions. Thus, workplace health promotion programs may inadvertently promote weight stigma and weight-based discrimination, particularly when they emphasize notions of individual responsibility.

Finally, Hardacre and Subašić investigate ways that leaders can mobilize support for gender equality. Across two experiments, they demonstrate that evoking a common cause increases women's collective action intentions and that male leaders invoke a higher sense of common cause and collective action intentions for both men and women, regardless of framing.

We hope that by bringing together research on the different ways that workplace inequality is manifested—across a range of different groups and social categories, and their intersection—we can shed greater light on this issue. From the papers included in this Research Topic, it is clear that there can be important similarities and differences in the psychological processes that are relevant to understanding how discrimination will impact on different targets. Ultimately, we hope this examination can lead toward interventions that are more nuanced and better able to combat inequalities in the workplace and in society more broadly.

## Author Contributions

All authors listed have made a substantial, direct and intellectual contribution to the work, and approved it for publication.

## Conflict of Interest

The authors declare that the research was conducted in the absence of any commercial or financial relationships that could be construed as a potential conflict of interest.
